# *Tetragenococcus halophilus* Alleviates Intestinal Inflammation in Mice by Altering Gut Microbiota and Regulating Dendritic Cell Activation via CD83

**DOI:** 10.3390/cells11121903

**Published:** 2022-06-12

**Authors:** S. M. Shamsul Islam, Hye-Myung Ryu, Seonghyang Sohn

**Affiliations:** 1 Department of Biomedical Science, Ajou University School of Medicine, Suwon 16499, Korea; shamsulislam21@gmail.com; 2 Department of Microbiology, Ajou University School of Medicine, Suwon 16499, Korea; naya101@hanmail.net

**Keywords:** ulcerative colitis, *Tetragenococcus halophilus*, gut microbiome, probiotics, CD83, mouse model

## Abstract

Ulcerative colitis (UC) is one of the major subtypes of inflammatory bowel disease with unknown etiology. Probiotics have recently been introduced as a treatment for UC. *Tetragenococcus halophilus* (*T. halophilus*) is a lactic acid-producing bacterium that survives in environments with high salt concentrations, though little is known about its immunomodulatory function as a probiotic. The purpose of this study is to determine whether *T. halophilus* exerts an anti-inflammatory effect on intestinal inflammation in mice. Colitis was induced in C57BL/6J mice by feeding 4% DSS in drinking water for 7 days. *T. halophilus* was orally administered with DSS. Anti-inflammatory functions were subsequently evaluated by flow cytometry, qRT-PCT, and ELISA. Gut microbial composition was analyzed by 16S rRNA metagenomic analysis. DSS-induced colitis mice treated with *T. halophilus* showed less weight loss and significantly suppressed colonic shortening compared to DSS-induced colitis mice. *T. halophilus* significantly reduced the frequency of the dendritic cell activation molecule CD83 in peripheral blood leukocytes and intestinal epithelial lymphocytes. Frequencies of CD8+NK1.1+ cells decreased in mice with colitis after *T. halophilus* treatment and IL-1β levels were also reduced. Alteration of gut microbiota was observed in mice with colitis after administration of *T. halophilus*. These results suggest *T. halophilus* is effective in alleviating DSS-induced colitis in mice by altering immune regulation and gut microbiome compositions.

## 1. Introduction

Inflammatory Bowel Disease (IBD), a chronic, recurrent inflammatory disorder of the intestine, can affect any part of the gastrointestinal tract. IBD can be divided into two main types: Crohn’s disease (CD) and ulcerative colitis (UC) [1]. The exact etiology of UC remains unclear. Immune dysregulation, abnormal immune response to luminal bacteria, and dysbiosis might be important factors [2]. In dextran sulfate sodium (DSS)-induced colitis models, adoptive transfer of bone marrow-derived dendritic cells (BM-DCs) can exacerbate symptoms [3], suggesting DCs play a key role in the immune balance of gut mucosa. CD83 is a glycosylated Ig-like type 1 membrane protein expressed as a surface marker for activated DCs in peripheral blood [4]. A significant number of DCs expressing CD83 molecules have been observed in CD patients [5] and murine colitis models [6]. Soluble CD83 has been suggested to have therapeutic and immunosuppressive properties by suppressing DCs-mediated T cell activation and ameliorating experimental colitis in mice [6].

Gut microbiotas play an important role in maintaining human health. They are also involved in the progression and development of human diseases [7]. UC is associated with altered compositions of the gut microbiota and mucosal immunity, resulting in excessive intestinal inflammation [8]. Evidence suggests reduced diversity in fecal microbiota is the most consistent indicator of UC [9]. The use of probiotics has been proposed to have beneficial effects on IBD due to the role of the microbiome in IBD pathogenesis [10]. Interaction of gut DCs with gut microflora can trigger immune modulation [11]. Lactic acid bacteria (LAB) can produce lactic acid as a major metabolite that can stimulate innate immunity and intestinal microbiota balancing at the mucosal site [12,13]. *Tetragenococcus halophilus* (*T. halophilus*), which is a gram-positive LAB and survives in a high-salt environment, is involved in the fermentation process of soy sauce and fish sauce. Until now, very few studies have focused on the immunological impact of *T. halophilus*. Thus, the aim of the present study was to investigate the anti-inflammatory effects of *T. halophilus* in DSS-induced colitis mice by evaluating its impact on immunomodulatory properties and the association with the gut microbiota.

## 2. Materials and Methods

### 2.1. Animals

Four-week-old C57BL/6J mice were purchased from Charles Rivers Laboratories (Yokohama, Japan). Animals were maintained until eight weeks old in a Specific Pathogen Free (SPF) environment at a temperature of 20–22 °C with a light-dark cycle of 12 h–12 h. Water and food were provided in a sterile environment. All studies were approved by Ajou University Institutional Animal Care and Use Committee (approval number: AMC-2021-0060). All animal experiments were performed according to the guidelines.

### 2.2. Dextran Sulphate Sodium (DSS) Induced Colitis Model Mice and Experimental Groups

Eight-week-old normal healthy mice were randomly assigned into four different groups (four mice in each group). In group 1, normal control mice received daily drinking water for 10 consecutive days. In group 2, normal mice were treated with 4.8 × 10^8^ CFU/mouse/day of *T. halophilus* for 10 consecutive days. In group 3, normal mice were given 4% DSS (molecular weight: 36–50 kDa; MP Biomedicals, LLC, Canada) with drinking water for 7 days for colitis induction and then without DSS for 3 days. In group 4, normal mice received 4% DSS with drinking water for 7 days, concurrently with 4.8 × 10^8^ CFU/mouse/day of *T. halophilus* orally daily, and 4.8 × 10^8^ CFU/mouse/day of *T. halophilus* orally daily without DSS for the next 3 days. *T. halophilus* was administered using an oral feeding cannula. Body weight, presence of blood in feces, and stool consistency were assessed daily, and a score was assigned to each of these parameters according to previously proposed criteria (Table 1). These scores were used to calculate the average daily disease activity index (DAI) [14]. Mice were euthanized on day 11 for further experiments.

### 2.3. Histopathological Examination

Distal colonic sections were fixed in 4% paraformaldehyde, embedded in paraffin, and cut into 6 µm-thick sections with a microtome (Reichert-Jung Biocut 2030, Ramsey, MN, USA). These sections were then stained with hematoxylin and eosin (H&E) to analyze histological changes.

### 2.4. Bacterial Strain and Culture Conditions

*T. halophilus* was purchased from Korean Collection for Type Cultures (KCTC). For bacterial growth, *T. halophilus* was cultured facultative anaerobically at 30 °C for 3–4 days using an MRS medium supplemented with 6.5% NaCl.

### 2.5. Intestinal Epithelial Lymphocytes (IELs) Isolation

Intestinal epithelial lymphocytes (IELs) were isolated as described [15]. Mice were euthanized, and the large intestine was gently removed from the stomach using scissors; fat and connective tissues were removed, and the intestine was washed three times with ice-cold PBS to remove intestinal contents. The intestines were opened longitudinally, cut into small pieces, and transferred to an RPMI medium containing 5% DTT, 0.5M EDTA, and 2% FBS for 1 h at 37 °C. Collected samples were vortexed for 30 s and passed through a 70 µm cell strainer and then incubated with collagenase I/dispase II digestion buffer for 30 min at 37 °C. Lymphocytes were collected from the supernatant and then subjected to flow cytometry analysis.

### 2.6. Flow Cytometry Analysis

Leukocytes were isolated from peripheral blood (PBL). Peritoneal macrophages (pMQ) were isolated from the peritoneal cavity. IELs were isolated from the intestinal epithelium. They were then stained with anti-mouse CD40, CD83, CD80, and CD86 antibodies at 4 °C for 30 min in the dark. PBL and splenocytes were stained with CD4, CD8, CD11b, Ly6G, and NK1.1 antibodies at 4 °C for 30 min in the dark. Cells isolated from lymph nodes (LN) were stained with CD4, CD8, and NK1.1 antibodies in the dark at 4 °C for 30 min. For intracytoplasmic staining of IELs, cells were incubated with brefeldin A (10 μg/mL) in RPMI media in a 37 °C incubator supplemented with 5% CO_2_ for 4 h. After incubation, cells were stained with CD4 and CD8 antibodies in the dark at 4 °C for 30 min. After washing, cells were fixed with an intracytoplasmic fixation buffer for 40 min at room temperature. After washing with a permeabilization buffer, cells were stained with interleukin (IL) -4 and IL-10 antibodies at room temperature for 40 min. The stained cells were analyzed by gating over 10,000 cells with a FACS Aria III flow cytometer (Becton Dickinson, San Jose, CA, USA). Sources of antibodies used in flow cytometry analysis are listed in Supplementary Table S1 (ST1).

### 2.7. Construction of 16S rRNA V3 and V4 Amplicon Sequencing Library

Fresh feces from mice were collected and analyzed for murine gut microbiota based on a 16S rRNA metagenomic sequence. In brief, the 16S rRNA gene sequencing of V3 and V4 amplicons were performed using 16S rRNA gene PCR primers (Forward Primer 5′-TCG TCG GCA GCG TCA GAT GTG TAT AAG AGA CAG CCT ACG GGN GGC WGC AG-3′, Reverse Primer 5′-GTC TCG TGG GCT CGG AGA TGT GTA TAA GAG ACA GGA CTA CHV GGG TAT CTA ATC C-3′). The libraries were sequenced on the Illumina HiSeq 2500 instrument (Illumina, St. Diego, CA, USA) (2 × 250 paired-end sequencing). Illumina adapter overhang nucleotide sequences (full-length primer sequence) were added to gene-specific sequences. At first, the adaptor sequences were removed from the original paired-end reads using CutAdapt v1.11. Secondly, the merged reads were produced with the first processed paired-end reads using FLASH v1.2.11. Then, low-quality merged reads were filtered out according to the following criteria; the read contained two or more ambiguous nucleotides, the average quality score was less than 20, and the reads’ length would be shorter than 300 bp after trimming low-quality bases. Finally, the potential chimeric reads were removed using ChimeraSlayer.

### 2.8. Taxonomy Profiling, OTUs, Alpha-Diversity, and Beta-Diversity

To calculate taxonomic abundance, consensus sequences were clustered using the following cd-hit v4.6 parameters: identification > 99% and coverage > 80%. Consensus sequences were then aligned to the nucleotide database from NCBI using the MegaBlast algorithm. Finally, taxonomic profiling was performed using National Center for Biotechnology Information (NCBI) taxonomy information and KronaTools. The number of operational taxonomic units (OTU) was determined by clustering the sequence of each sample having a 97% sequence identity with cutoff using Quantitative Insights for Microbial Ecology (QIIME) software (v.1.8.0). To measure beta-diversity, the Bray–Curtis distance was applied by identifying differences between organism compositions. Principal component analysis (PCA) was then performed using beta diversity results.

### 2.9. Quantitative Reverse Transcription Polymerase Chain Reaction (qRT-PCR)

Total RNA was isolated from mice PBL and splenocytes using TRIzol reagent (Thermo Fisher, Waltham, MA, USA) following the manufacturer’s recommendations. RNA was quantified with a NanoDrop spectrophotometer (NanoDrop Technologies, Wilmington, NC, USA) and reverse transcribed into cDNA using a PrimeScript cDNA Synthesis kit (Takara Shuzo Co., Otsu, Shiga, Japan). qRT-PCR was performed in duplicate for each target transcript using SYBR green PCR Master Mix (Applied Biosystems, Foster City, CA, USA) and gene-specific primers on a 7500 Real-Time PCR System (Applied Biosystems, Waltham, MA, USA). For qRT-PCR, 1 µL cDNA was used as a template. The final reaction volume was 20 µL. Each reaction contained gene-specific primers listed in Supplementary Table S2 (ST2). The PCR reaction was performed with pre-denaturation at 50 °C for 2 min and 95 °C for 10 min followed by 40 cycles at 95 °C for 15 s and 60 °C for 1 min. Relative gene expression levels were calculated using the 2^−ΔΔCt^ method [16]. The expression level of each gene was normalized to the level of β-actin, a housekeeping gene. Data are expressed as fold change compared to untreated control.

### 2.10. Enzyme-Linked Immunosorbent Assay (ELISA)

After sacrifice, blood was collected from the heart of each mouse. Plasma levels of IL-1β and TNFα were analyzed using a commercial ELISA kit (R&D Systems, Minneapolis, MN, USA) according to the manufacturer’s instructions. Mouse plasma samples were diluted 2 folds for assay. Absorbance values of samples were read at a wavelength of 450 nm using a Bio-Rad model 170-6850 microplate reader (Bio-Rad, Hercules, CA, USA). ELISA was repeated in duplicate wells.

### 2.11. Statistical Analysis

Statistical differences between experimental groups were determined using one-way ANOVA, followed by Bonferroni’s multiple comparison test with GraphPad Prism version 8.3.1 for Windows (GraphPad Software, La Jolla, CA, USA). The Mann–Whitney U test was used to compare the differences between the two groups. Statistical significance was considered when the *p*-value was less than 0.05.

## 3. Results

### 3.1. Administration of T. halophilus Prevents DSS-Induced Weight Loss and Colon Damage in Mice

**Figure 1:**
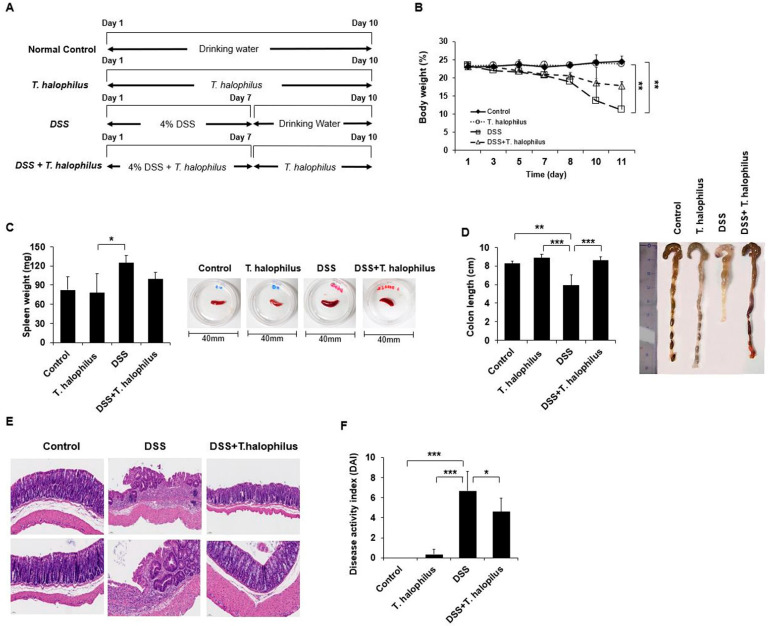
Effects of *T. halophilus* in DSS-induced colitis mice. (**A**) Schematic diagram of mice treatment and group distribution. (**B**) Effect of *T. halophilus* administration on weight change of mice after colitis induction. (**C**) Changes in spleen weights of mice after colitis induction. (**D**) Changes in colon lengths after administration of *T. halophilus*. (**E**,**F**) Colon histology and disease activity index (DAI) in mice with DSS-induced colitis after administration of *T. halophilus*. For statistical analysis, a one-way ANOVA followed by Bonferroni’s multiple comparison test was performed with GraphPad. The number of mice used for experiments was four in each group. Significantly different *p*-values are indicated by an asterisk as follows: *, *p* < 0.05; **, *p* < 0.01; ***, *p* < 0.001.

For colitis induction, mice were treated with 4% DSS with drinking water for 7 days with or without *T. halophilus* for 10 consecutive days. Control groups were treated with and without *T. halophilus* for 10 consecutive days (Figure 1A). Mice body weight was measured every 2 days. Colon length and spleen weight were quantified the day after the last doses. Body weights of DSS-induced colitis mice were significantly decreased compared to *T. halophilus* treated normal mice group (11.22 ± 7.72% vs. 23.78 ± 1.08%, *p* < 0.01) and normal control mice (11.22 ± 7.72% vs. 24.44 ± 1.53%, *p* < 0.01) (Figure 1B). *T. halophilus* administered colitis mice had less weight loss compared to colitis mice, although the difference between the two was not statistically significant (11.22 ± 7.72% vs. 17.88 ± 1.03%) (Figure 1B). Spleen weights of DSS-induced colitis mice were increased compared to those of normal mice treated with *T. halophilus* (124.75 ± 10.43 mg vs. 78.00 ± 30.15 mg, *p* < 0.05). (Figure 1C). DSS-treated mice had shorter colon lengths compared to normal control mice (5.92 ± 1.14 cm vs. 8.22 ± 0.30 cm, *p* < 0.01) and normal mice treated with *T. halophilus* (5.92 ± 1.14 cm vs. 8.86 ± 0.37 cm, *p* < 0.001). The shortened colon length was significantly alleviated by *T. halophilus* treatment in colitis mice (5.92 ± 1.03 cm vs. 8.57 ± 0.43 cm, *p* < 0.001) (Figure 1D).

### 3.2. Administration of T. halophilus Ameliorates DSS-Induced Colitis and Reduces Disease Activity Index

Clinical symptoms of colitis over time in DSS-induced colitis mice were evaluated. These symptoms include weight loss, diarrhea, blood in stool, and death [17]. DSS-induced colitis mice had higher disease activity index (DAI) scores compared to *T. halophilus* treated colitis mice, indicating *T. halophilus* was able to prevent or ameliorate colitis symptoms in mice (Figure 1F, Table 1). Histological examination showed extensive infiltration of inflammatory cells into the colonic tissues of colitis mice (Figure 1E). The lesions in colitis mice were reduced by treatment with *T. halophilus*. Although *T. halophilus* treated colitis mice showed some inflammatory lesions, their severity was lower compared to non-treated colitis mice.

### 3.3. T. halophilus Affects the Activation of DCs in PBL and IELs of Mice with DSS-Induced Colitis

**Figure 2:**
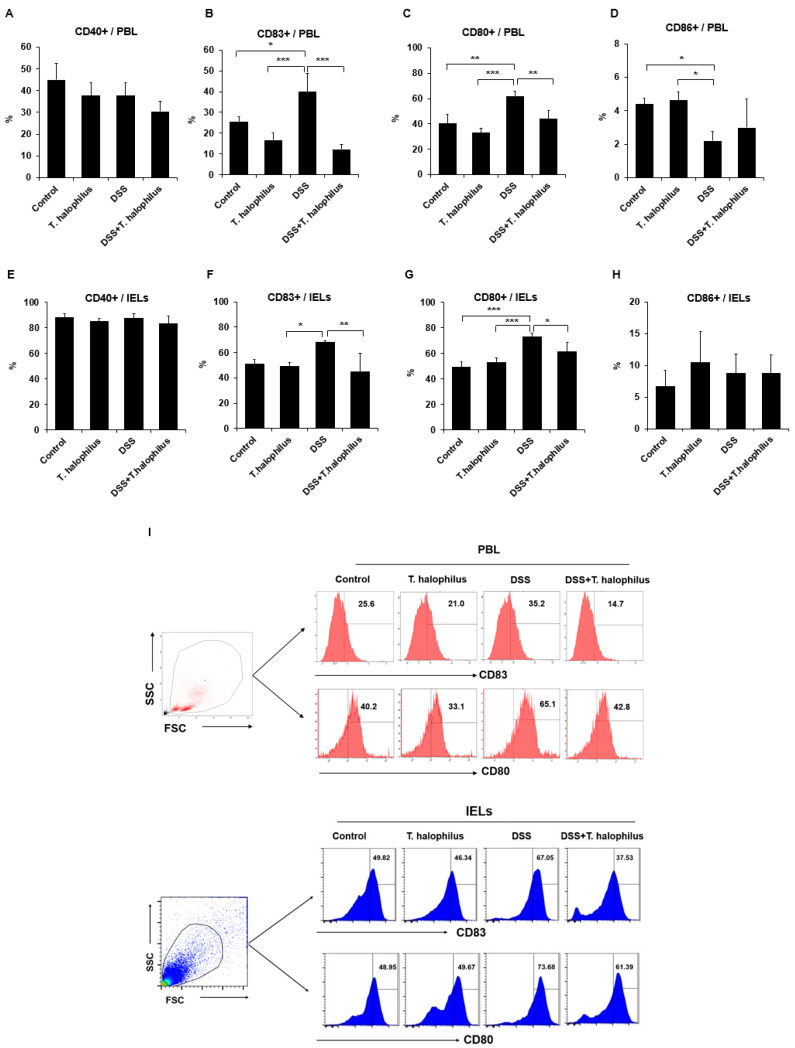
Frequencies of cells positive for DCs costimulatory molecules CD40, CD83, CD80, and CD86 in peripheral blood leukocytes (PBL) and intestinal epithelial lymphocytes (IELs) were assessed in normal control mice, normal mice treated with *T. halophilus* (4.8 × 10^8^ CFU/mouse/day), DSS-induced colitis mice, and colitis mice treated with *T. halophilus* (4.8 × 10^8^ CFU/mouse/day) (**A**–**H**). Isolated PBL and IELs were evaluated by flow cytometry surface staining. A representative histogram of CD83+ and CD80+ cells for PBL and IELs are shown in (**I**). For statistical analysis, a one-way ANOVA followed by Bonferroni’s multiple comparison test was performed with GraphPad. The number of mice used for the experiments was four in each group. Experiments were performed independently at least twice. Significantly different *p*-values are indicated by an asterisk as follows: *, *p* < 0.05; **, *p* < 0.01; ***, *p* < 0.001.

Frequencies of positive cells for DCs activating co-stimulatory molecules CD40, CD83, CD80, and CD86 in the PBL of mice were analyzed by a flow cytometer. Frequencies of CD83+ cells were significantly increased in colitis mice compared to normal control mice in PBL (39.88 ± 8.91% vs. 25.70 ± 2.36%, *p* < 0.05) and compared to normal mice treated with *T. halophilus* (39.88 ± 8.91% vs. 16.70 ± 3.60%, *p* < 0.001) (Figure 2B). The frequencies of CD83+ cells in colitis mice were also significantly increased compared with *T. halophilus* treated normal mice in IELs (67.92 ± 1.10% vs. 49.06 ± 3.11%, *p* < 0.001) (Figure 2F). Administration of *T. halophilus* to DSS-induced colitis mice significantly decreased the frequencies of CD83+ cells compared to non-treated colitis mice in PBL (12.33 ± 2.11% vs. 39.88 ± 8.91%, *p* < 0.001) and in IELs (44.63 ± 14.81% vs. 67.92 ± 1.10%, *p* < 0.01), respectively (Figure 2B,F). Frequencies of CD80+ cells were significantly increased in colitis mice compared to normal control mice in PBL (61.60 ± 4.38% vs. 40.22 ± 7.48%, *p* < 0.001) and in IELs (73.13 ± 2.67% vs. 49.40 ± 4.07%, *p* < 0.001), and also compared to normal mice treated with *T. halophilus* in PBL (61.60 ± 4.38% vs. 32.90 ± 3.76%, *p* < 0.001) and in IELs (73.13 ± 2.67% vs. 52.85 ± 3.81%, *p* < 0.001) (Figure 2C,G). The administration of *T. halophilus* to DSS-induced colitis mice significantly decreased the frequencies of CD80+ cells more than in non-treated colitis mice in PBL (43.95 ± 6.50 % vs. 61.60 ± 4.38%, *p* < 0.01) and in IELs (61.43 ± 7.20% vs. 73.13 ± 2.67%, *p* < 0.05), respectively (Figure 2C,G). Frequencies of CD86+ cells were significantly decreased in colitis mice compared to normal control mice (2.2 ± 0.57% vs. 4.4 ± 0.35%, *p* < 0.05) or normal mice treated with *T. halophilus* (2.2 ± 0.57% vs. 4.6 ± 0.49%, *p* < 0.05) in PBL, however, in IELs no significant differences were observed (Figure 2D,H). No significant differences were observed in the frequencies of CD40+ cells among the groups in PBL and IELs (Figure 2A,E). Representative histograms of CD83+ and CD80+ cells of PBL and IELs are shown in Figure 2I. Frequencies of positive cells for DCs activating co-stimulatory molecules CD40, CD83, CD80, and CD86 in pMQ were also assessed by flow cytometry analysis, but no significant differences were observed among the groups (Supplementary Figure S1).

### 3.4. Frequencies of CD8+NK1.1+ T Cells in DSS-Induced Colitis Mice

**Figure 3:**
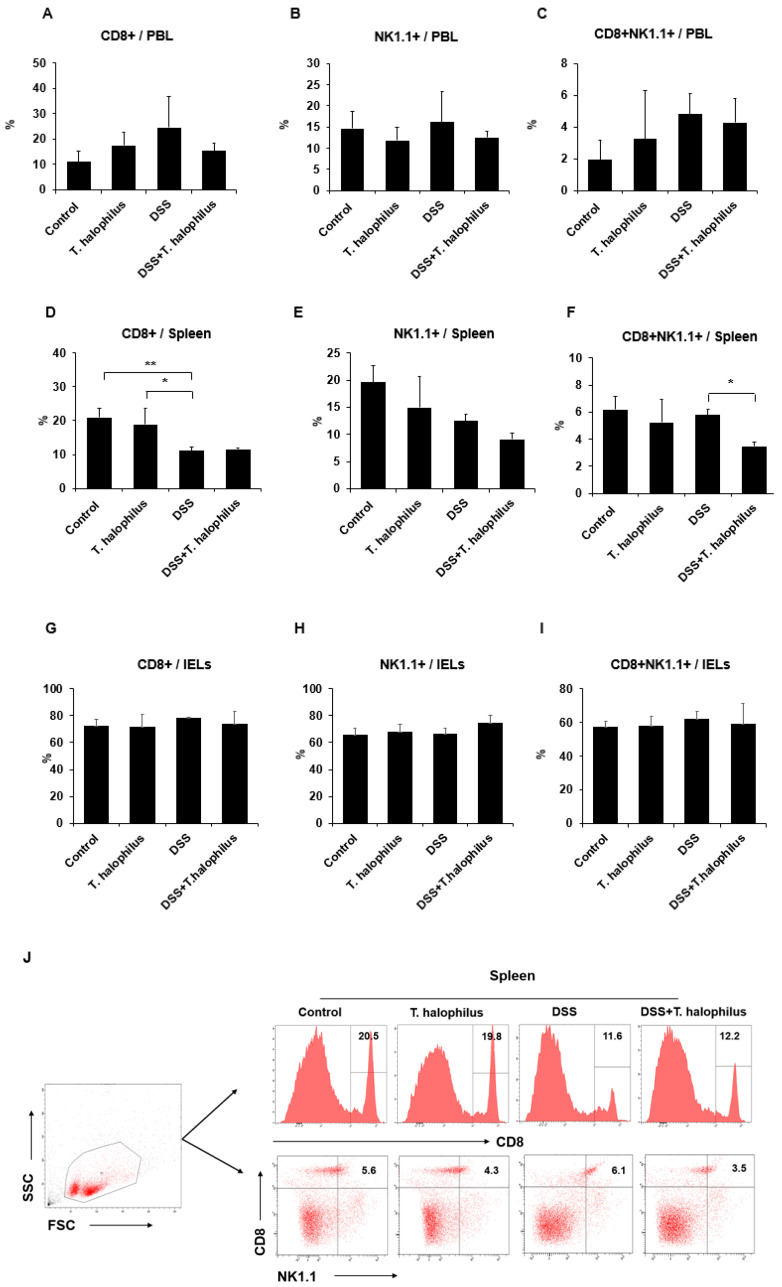
Effects of *T. halophilus* on the frequencies of CD8+NK1.1+ cells in DSS-induced colitis mice. Frequencies of CD8+, NK1.1+, and CD8+NK1.1+ cells in the surface of peripheral blood leukocytes (PBL), spleens, and intestinal epithelial cells (IELs) of normal control mice, normal mice treated with *T. halophilus* (4.8 × 10^8^ CFU/mouse/day), DSS-induced colitis mice, and colitis mice treated with *T. halophilus* (4.8 × 10^8^ CFU/mouse/day) for 10 consecutive days were evaluated by flow cytometric analysis (**A**–**I**). A representative histogram of CD8+ cells and dot plot of CD8+NK1.1+ cell frequencies in the spleen are shown in (**J**). The *p*-value was determined by one-way ANOVA followed by Bonferroni’s multiple comparison test with GraphPad. Experiments were performed more than two independent times and four mice were used in each group. Significantly different *p*-values are indicated by an asterisk as follows: *, *p* < 0.05; **, *p* < 0.01.

Frequencies of CD8+, NK1.1+, and CD8+NK1.1+ cells in PBL, splenocytes, and IELs of mice were analyzed using a flow cytometer. Frequencies of CD8+, NK1.1+, and CD8+NK1.1+ cells in PBL were not significantly different among groups (Figure 3A–C). However, in PBL, the frequencies of CD4+ cells were significantly decreased in DSS-induced colitis mice compared with normal mice treated with *T. halophilus* (18.72 ± 2.61% vs. 35.30 ± 6.84%, *p* < 0.05) (Supplementary Figure S2). In splenocytes, frequencies of CD8+ T cells were decreased in colitis mice compared to normal control mice (11.10 ± 1.25% vs. 20.90 ± 2.82%, *p* < 0.01) and normal mice treated with *T. halophilus* (11.10 ± 1.25% vs. 18.85 ± 3.14%, *p* < 0.05) (Figure 3D). In addition, the frequencies of CD4+ T cells in the splenocytes of DSS-induced colitis mice were also significantly decreased compared to normal control mice (11.30 ± 1.92% vs. 29.90 ± 4.14%, *p* < 0.01) or normal mice treated with *T. halophilus* (11.30 ± 1.92% vs. 28.57 ± 8.81%, *p* < 0.01) (Supplementary Figure S2). There were no significant differences observed in the frequencies of NK1.1+ cells among the groups (Figure 3E). However, administration of *T. halophilus* significantly decreased the frequencies of CD8+NK1.1+ cells in the splenocytes of DSS-induced colitis mice compared to non-treated colitis mice (3.47 ± 0.32% vs. 5.77 ± 0.45%, *p* < 0.05) (Figure 3F). There were no significant differences were observed among the groups in IELs (Figure 3G–I). There were no significant differences in frequencies of CD4+, CD8+, NK1.1+, or CD8+NK1.1+ cells in LN among groups (Supplementary Figure S3). Representative histograms of CD8+ cells and dot plots of CD8+NK1.1+ cells in the spleens are shown in Figure 3J.

### 3.5. Frequencies of Ly6G+ Neutrophils in T. halophilus-Treated Colitis Mice

**Figure 4:**
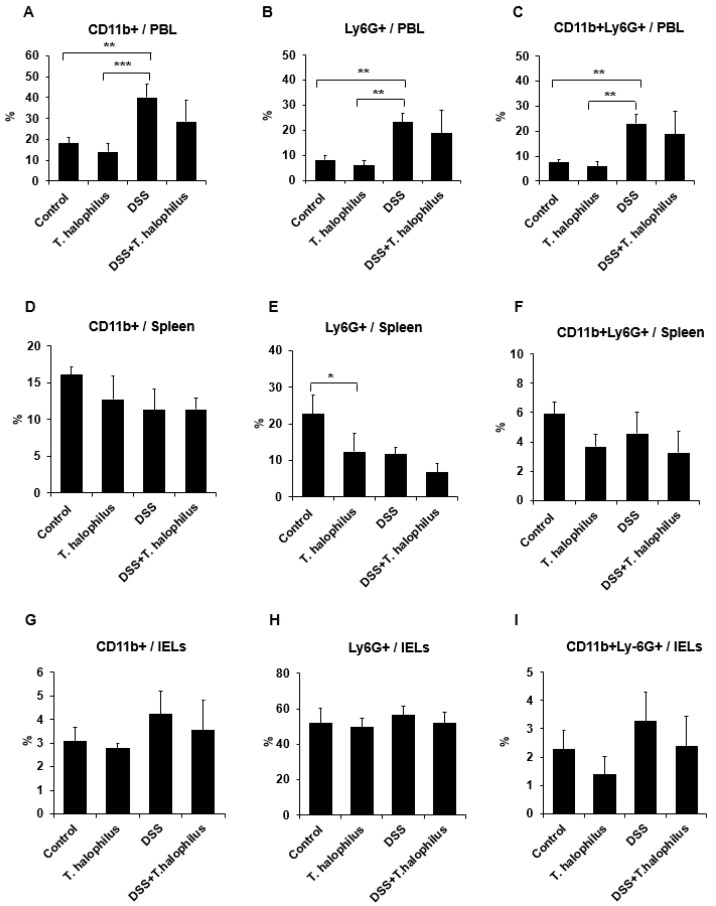

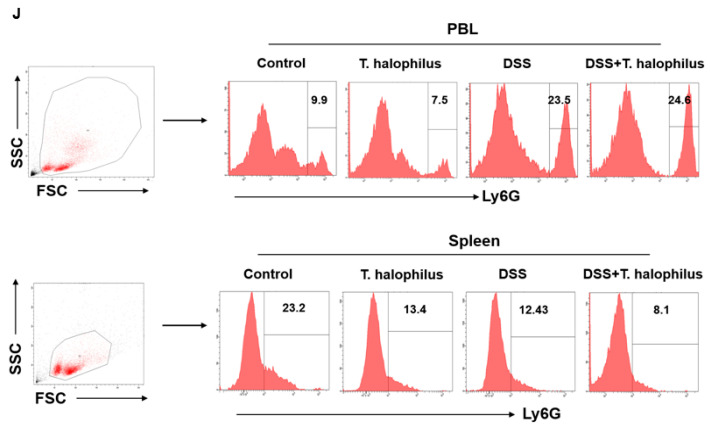
Effects of *T. halophilus* on Ly6G+ cells in DSS-induced colitis mice. Frequencies of CD11b+, Ly6G+, and CD11b+Ly6G+ cells in the surface of peripheral blood leukocytes (PBL) splenocytes, and intestinal epithelial cells (IELs) of normal control mice, normal mice treated with *T. halophilus* (4.8 × 10^8^ CFU/mouse/day), DSS-induced colitis mice, and colitis mice treated with *T. halophilus* (4.8 × 10^8^ CFU/mouse/day) for 10 consecutive days were evaluated by flow cytometric analysis (**A**–**I**). Representative histograms of Ly6G+ cell frequencies in PBL and spleen are shown in (**J**). One-way ANOVA followed by Bonferroni’s multiple comparison test was performed with GraphPad for statistical analysis. Four mice were used in each experimental group. Experiments were performed independently at least twice. Significantly different *p*-values are indicated by an asterisk as follows: *, *p* < 0.05; **, *p* < 0.01; ***, *p* < 0.001.

Frequencies of CD11b+, Ly6G+, and CD11b+Ly6G+ cells in PBL, splenocytes, and IELs of mice were analyzed by flow cytometry. Frequencies of CD11b+ cells in PBL of DSS-induced colitis mice were significantly increased compared to normal control mice (40.04 ± 6.51% vs. 18.20 ± 1.56%, *p* < 0.01) and normal mice treated with *T. halophilus* (40.04 ± 6.51% vs. 14.08 ± 3.84%, *p* < 0.001) (Figure 4A). Frequencies of neutrophils expressing Ly6G+ cells in PBL of colitis mice were significantly higher compared to normal control mice (23.15 ± 3.65% vs. 8.22 ± 1.56%, *p* < 0.01) and normal mice treated with *T. halophilus* (23.15 ± 3.65% vs. 5.95 ± 1.95%, *p* < 0.01) (Figure 4B). Additionally, frequencies of CD11b+Ly6G+ cells in the PBL of DSS-induced colitis mice were significantly increased compared to normal control mice (23.07 ± 3.57% vs. 7.52 ± 1.06%, *p* < 0.01) and normal mice treated with *T. halophilus* (23.07 ± 3.57% vs. 5.82 ± 1.93%, *p* < 0.01) (Figure 4C). In splenocytes, the administration of *T. halophilus* to normal mice significantly decreased frequencies of neutrophils expressing Ly6G+ cells compared to normal control mice treated with (12.40 ± 4.96% vs. 22.70 ± 5.07%, *p* < 0.05) (Figure 4E), however, no significant differences were observed in the frequencies of CD11b+ and CD11b+Ly6G+ cells among the groups (Figure 4D,F). There were no significant differences observed among the groups in IELs as well (Figure 4G–I). Representative histogram and dot plots of Ly6G+ cells in PBL and spleen are shown in Figure 4J.

### 3.6. T. halophilus Administration Affects Microbial Abundance in DSS-Induced Colitis Mice at Phylum Level

**Figure 5:**
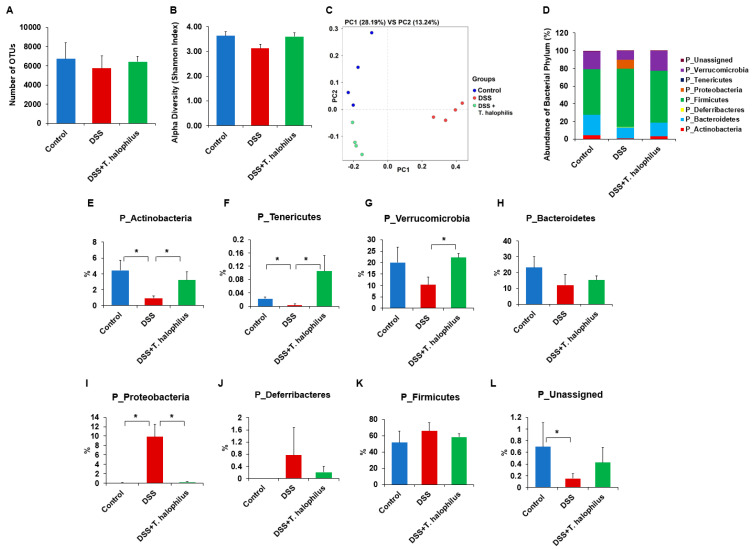

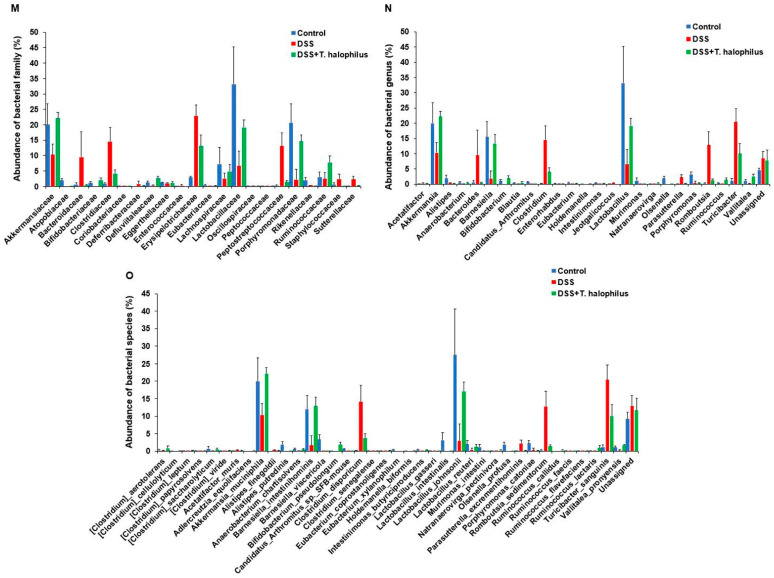
*T. halophilus* modulates gut microbiota compositions in DSS-induced colitis mice. Fecal samples were collected from normal control mice, 4% DSS-induced colitis mice, and colitis mice treated with *T. halophilus*. Microbiome analysis was performed using sequencing data of 16S rRNA V3 and V4 amplicons. (**A**) Operational *Taxonomic* Units (OTUs), (**B**) Alpha diversity (Shannon index), and (**C**) Principal component analysis (PCA) plot. (**D**–**L**) Abundance of bacterial phyla. Abundance of bacterial (**M**) family, (**N**) genus, and (**O**) species showed significant differences among groups. Four mice were used in each experimental group. The Mann–Whitney U test was performed with GraphPad for statistical analysis. *, *p* < 0.05.

Fresh fecal samples were collected from normal control mice, DSS-induced colitis mice, and colitis mice treated with *T. halophilus*. V3-V4 regions of 16S rRNA of all samples were amplified. No significant differences were observed in terms of alpha diversity (Figure 5B) or OUTs (Figure 5A). Beta diversity analysis showed changes in species diversity and disease association along with principal components analysis (PCA) 1 and 2 (Figure 5C). Figure 5D shows the abundance of the bacterial phylum. At the bacterial phylum level, *Actinobacteria* (*p* < 0.05) and *Tenericutes* (*p* < 0.03) showed reduced abundance in colitis mice compared to normal control mice (Figure 5E,F), whereas *Proteobacteria* (*p* < 0.03) showed increased abundance in colitis mice compared to normal control mice (Figure 5I). *T. halophilus* administration to colitis mice significantly reduced the abundance of *Proteobacteria* (*p* < 0.03, Figure 5I) and increased the population of *Actinobacteria* (*p* < 0.05, Figure 5E), *Tenericutes* (*p* < 0.03, Figure 5F), and *Verrucomicrobia* (*p* < 0.04, Figure 5G). Although *Bacteroidetes* and *Firmicutes* are the most abundant population in phyla, no significant difference was observed between the groups (Figure 5H,K), and no significant difference in phylum *Deferribacteres* was observed between the groups (Figure 5J). However, a significant difference in unassigned bacterial phylum was observed between normal control mice and non-treated colitis mice (*p* < 0.03, Figure 5L). The abundance of bacterial phylum is shown in Supplementary Table S3 (ST3).

### 3.7. T. halophilus Administration Affects Microbial Abundance in DSS-Induced Colitis at Family and Genus Levels

At the family level, *Bifidobacteriaceae* (*p* < 0.03), *Defluviitaleaceae* (*p* < 0.03), *Eubacteriaceae* (*p* < 0.03), *Lactobacillaceae* (*p* < 0.03), *Porphyromonadaceae* (*p* < 0.03), and *Rikenellaceae* (*p* < 0.03) showed significantly reduced abundance in colitis mice compared to normal control mice (Figure 5M). *Clostridiaceae* (*p* < 0.03), *Erysipelotrichaceae* (*p* < 0.03), *Peptostreptococcaceae* (*p* < 0.03), and *Sutterellaceae* (*p* < 0.03) showed increased abundance in colitis mice compared to normal controls (Figure 5M). *T. halophilus* administration to colitis mice increased *Akkermansiaceae* (*p* < 0.03), *Defluviitaleaceae* (*p* < 0.03), *Eubacteriaceae* (*p* < 0.03), *Lactobacillaceae* (*p* < 0.03), *Porphyromonadaceae* (*p* < 0.03), and *Ruminococcaceae* (*p* < 0.03), and reduced *Clostridiaceae* (*p* < 0.03), *Erysipelotrichaceae* (*p* < 0.03), *Peptostreptococcaceae* (*p* < 0.03), *Staphylococcaceae* (*p* < 0.03), and *Sutterellaceae* (*p* < 0.03) (Figure 5M). At the genus level, colitis mice showed increased populations of *Acetatifactor* (*p* < 0.03), *Clostridium* (*p* < 0.03), *Jeotgalicoccus* (*p* < 0.03), *Parasutterella* (*p* < 0.03), *Romboutsia* (*p* < 0.03), and *Turicibacter* (*p* < 0.03), and reduced populations of *Alistipes* (*p* < 0.03), *Anaerobacterium* (*p* < 0.03), *Barnesiella* (*p* < 0.03), *Eubacterium* (*p* < 0.03), *Lactobacillus* (*p* < 0.03), *Natranaerovirga* (*p* < 0.03), *Porphyromonas* (*p* < 0.03), *Ruminococcus* (*p* < 0.03), and *Vallitalea* (p < 0.03) compared to normal controls (Figure 5N). *T. halophilus* treatment in colitis mice increased *Akkermansia* (*p* < 0.03), *Anaerobacterium* (*p* < 0.03), *Barnesiella* (*p* < 0.003), *Eubacterium* (*p* < 0.03), *Lactobacillus* (*p* < 0.03), *Natranaerovirga* (*p* < 0.03), *Ruminococcus* (*p* < 0.03), and *Vallitalea* (*p* < 0.03), and decreased *Clostridium* (*p* < 0.03), *Parasutterella* (*p* < 0.03), *Romboutsia* (*p* < 0.03), *Staphylococcus* (*p* < 0.03), and *Turicibacter* (*p* < 0.03) compared to untreated colitis mice (Figure 5N). The abundance of bacterial family and genus are shown in Supplementary Table S3 (ST3).

### 3.8. T. halophilus Administration Affects Microbial Abundance in DSS-Induced Colitis at Species Level

At the species level, DSS-induced colitis mice showed increased populations of *Acetatifactor muris* (*p* < 0.03), *Clostridium disporicum* (*p* < 0.03), *Parasutterella excrementihominis* (*p* < 0.03), *Romboutsia sedimentorum* (*p* < 0.03), and *Turicibacter sanguinis* (*p* < 0.03), and decreased populations of *Clostridium leptum* (*p* < 0.04), *Anaerobacterium chartisolvens* (*p* < 0.03), *Barnesiella intestinihominis* (*p* < 0.03), *Barnesiella viscericola* (*p* < 0.03), *Intestinimonas butyriciproducens* (*p* < 0.03), *Lactobacillus gasseri* (*p* < 0.03), *Lactobacillus johnsonii* (*p* < 0.03), *Natranaerovirga pectinivora* (*p* < 0.03), *Porphyromonas catoniae* (*p* < 0.03), *Ruminococcus faecis* (*p* < 0.03), and *Vallitalea pronyensis* (*p* < 0.03) compared to normal controls (Figure 5O). Administration of *T. halophilus* reduced *Alistipes finegoldii* (*p* < 0.04), *Clostridium disporicum* (*p* < 0.03), *Parasutterella excrementihominis* (*p* < 0.03), *Romboutsia sedimentorum* (*p* < 0.03), and *Turicibacter sanguinis* (*p* < 0.03), and increased bacterial populations of *Clostridium saccharolyticum* (*p* < 0.03), *Akkermansia muciniphila* (*p* < 0.03), *Anaerobacterium chartisolvens* (*p* < 0.03), *Barnesiella intestinihominis* (*p* < 0.03), *Intestinimonas butyriciproducens* (*p* < 0.03), *Lactobacillus gasseri* (*p* < 0.04), *Lactobacillus johnsonii* (*p* < 0.03), and *Ruminococcus lactaris* (*p* < 0.03) in colitis mice (Figure 5O). The abundance of bacterial species is shown in Supplementary Table S3 (ST3).

### 3.9. T. halophilus Administration Increased IL-4+ and IL-10+ T Cells in Intestinal Epithelial Lymphocytes (IELs)

**Figure 6:**
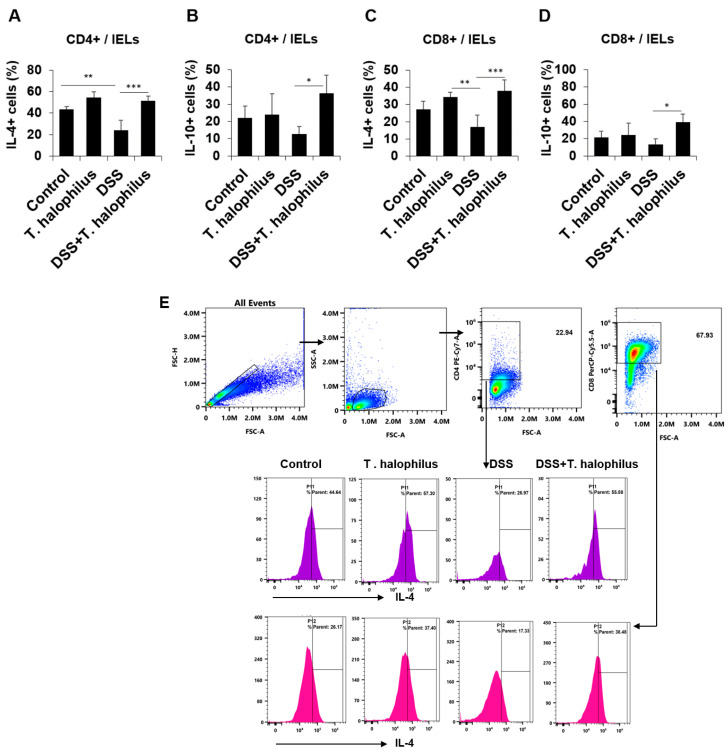
Frequencies of IL-4+ and IL-10+ cells in CD4+ and CD8+ T cells from intestinal epithelial lymphocytes (IELs) of normal control mice, normal mice treated with *T. halophilus*, DSS-induced colitis mice, and colitis mice treated with *T. halophilus* were evaluated by flow cytometry analysis (**A**–**D**). The gating strategy of IL-4 in IELs is shown in (**E**). A one-way ANOVA followed by Bonferroni’s multiple comparison test was performed with GraphPad for statistical analysis. Four mice were used in each experimental group. Experiments were performed in duplicate. Significantly different *p*-values are indicated with asterisks as follows: *, *p* < 0.05; **, *p* < 0.01; ***, *p* < 0.001.

The frequencies of IL-4 and IL-10 cytokines in CD4+ and CD8+ T cells of the IELs were analyzed by flow cytometry. IL-4+ cells in CD4+ T cells from DSS-induced colitis mice were significantly reduced compared to controls (23.96 ± 9.40% vs. 43.38 ± 2.55%, *p* < 0.01) (Figure 6A). Administration of *T. halophilus* in mice with DSS-induced colitis upregulated the frequencies of IL-4+ cells in CD4+ T cells (51.27 ± 4.58% vs. 23.96 ± 9.40%, *p* < 0.001) and in CD8+ T cells (37.27 ± 90% vs. 16.93 ± 6.88%, *p* < 0.001) (Figure 6A,C). The administration of *T. halophilus* to DSS-induced colitis mice also increased the frequencies of IL-10+ cells in CD4+ T cells (36.31 ± 10.62% vs. 12.61 ± 4.45%, *p* < 0.05) and CD8+ T cells (38.97 ± 9.79% vs. 13.26 ± 6.74%, *p* < 0.05) (Figure 6B,D). Representative gating strategies of IL-4 in IELs are shown in (Figure 6E).

### 3.10. T. halophilus Administration Downregulates IL-1β and TNFα in DSS-Induced Colitis Mice

**Figure 7:**
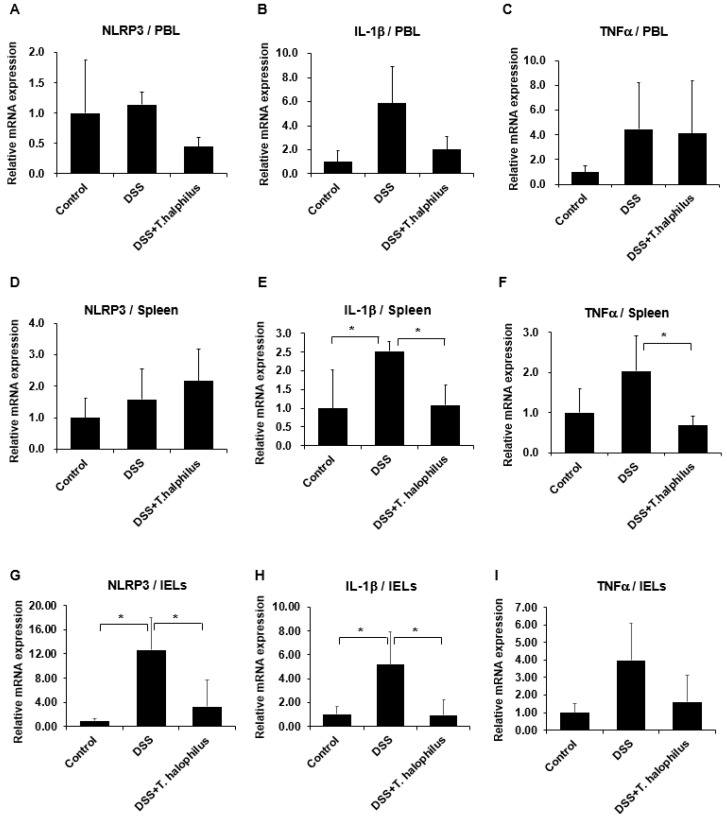
Effects of *T. halophilus* on mRNA expression levels of NLRP3, IL-1β, and TNFα in peripheral blood leukocytes (PBL), spleens, and intestinal epithelial lymphocytes (IELs) were quantified by real-time PCR (**A**–**I**). Four mice were used in each experimental group. The experiment was conducted in duplicate. Significantly different *p*-values are indicated by an asterisk as follows: *, *p* < 0.05.

The mRNA expression levels of NLRP3, IL-1β, and TNFα were analyzed using PBL, splenocytes, and in IELs. After *T. halophilus* administration to DSS-induced colitis mice, IL-1β was significantly downregulated in the spleen (*p* < 0.05) and in IELs (*p* < 0.05), respectively (Figure 7E,H), but no significant differences were observed in NLRP3 in PBL and splenocytes among the groups (Figure 7A,D), however, NLRP3 was significantly decreased in IELs (*p* < 0.05) of DSS-induced colitis mice after *T. halophilus* administration (Figure 7G). In PBL, no significant differences were observed in the mRNA expression levels of NLRP3, IL-1β, and TNFα, (Figure 7A–C), but the TNFα mRNA level was significantly decreased in splenocytes (*p* < 0.05) after *T. halophilus* administration to DSS-induced colitis mice (Figure 7F), although no significant difference was observed in IELs (Figure 7I).

### 3.11. T. halophilus Administration Reduces Plasma Levels of IL-1β in DSS-Induced Colitis Mice

**Figure 8:**
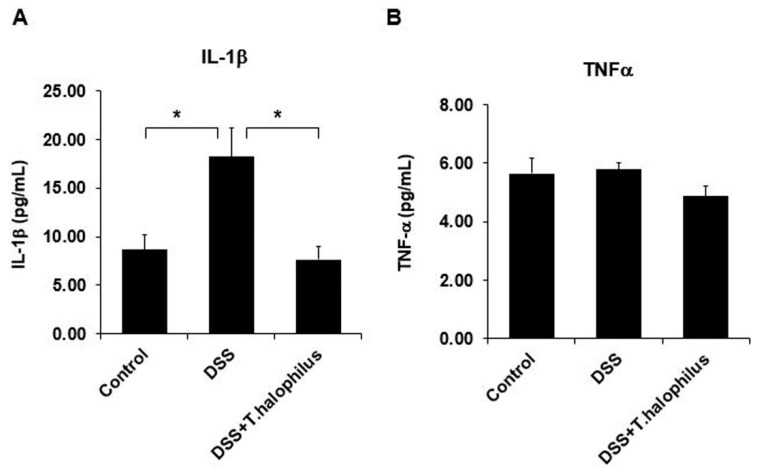
Effects of *T. halophilus* on plasma cytokine levels in DSS-induced colitis mice. Plasma IL-1β and TNFα levels in normal control mice, DSS-induced colitis mice, and colitis mice treated with *T. halophilus* were analyzed by ELISA (**A**,**B**). Four mice were used in each experimental group. The experiment was performed in duplicate. Significantly different *p*-values are indicated by an asterisk as follows: *, *p* < 0.05.

Cytokine levels of IL-1β and TNFα in plasma samples were determined by ELISA. The IL-1β concentration was significantly higher in colitis mice compared to normal control mice (18.21 ± 3.01 pg/mL vs. 8.65 ± 1.54 pg/mL, *p* < 0.05). *T. halophilus* administration significantly downregulated IL-1β levels in colitis mice (7.65 ± 1.35 pg/mL vs. 18.21 ± 3.01 pg/mL, *p* < 0.05) compared to untreated colitis mice (Figure 8A). TNFα levels were not different between normal control mice and colitis mice. *T. halophilus* administration to colitis mice reduced TNFα levels but was not statistically significant (Figure 8B).

## 4. Discussion

We provided evidence for the ameliorative ability of *T. halophilus* to protect against intestinal inflammatory damage induced by DSS in colitis model mice. When DSS reaches the intestinal lumen, it acts as a toxin that destroys the integrity of the colonic mucosa, increases intestinal permeability, and exacerbates the local and systemic inflammatory response [18]. In our study, administration of *T. halophilus* down-regulated the disease severity index and restoration of gut microbiota. In addition, the immunomodulatory properties of *T. halophilus* seem to help alleviate intestinal inflammation in mice.

Administration of DSS to mice results in clinical and histopathological characteristics similar to those seen in human IBD [17]. In our study, the administration of DSS to mice leads to several clinical manifestations compared to normal mice, including weight loss, diarrhea, bloody stool, colon shorting, as well as spleen enlargement. Administration of *T. halophilus* reduced these clinical symptoms and alleviates colitis. *T. halophilus* can survive for years in up to 30% salinity during the fermentation process that makes anchovy sauce, soybean paste, red pepper paste [19], or cheese [20]. Until now, there have been few reports of the therapeutic effects of bacteria surviving in high-salinity environments such as *T. halophilus* in disease models. This is the first report on its anti-inflammatory function in models of inflammatory bowel diseases. The high vitality of *T. halophilus*, which live in high salinity and survives in bile salts and extremely low pH, can survive passing through the stomach and reach the intestine [19]. Administration of probiotics can modulate the action of DCs to produce IL-10 and IL-12 along with the expression of co-stimulatory molecules [21], and modify Th1/Th2 cytokine production in various autoimmune diseases [22].

According to Grosche et al., CD83 plays an important role in controlling and maintaining immune tolerance [23]. In IBD patients, accidental activation of CD83 by microbial antigen induces Th1 and Th17 cell’s immune responses in intestinal tissue. This response is characterized by the release of excessive amounts of pro-inflammatory cytokines along with the activation of macrophages and granulocytes that promote inflammation [24]. Downregulation of CD83 by *T. halophilus* administration can be explained by bacterial specificity. According to Amar et al., it was reported that the expression levels of CD80 and CD83 differ depending on the type of bacteria [25]. The regulation of CD83 and CD80 appears to be more affected by *T. halophilus* than other costimulatory molecules. CD83 is known to be expressed at higher levels in UC patients [26], and our present study shows inhibition of CD83 through administration of *T. halophilus* and amelioration of inflammation are correlated.

It has also been reported *T. halophilus* significantly augmented the gene expressions of IL-10 [13], and IL-10 deficient mice have been used as a model reflecting the characteristics of colitis [27]. Increased IL-10 secretion reduces pro-inflammatory cytokines and prevents cell damage [28]. *T. halophilus* Strain Th221 provided a therapeutic effect in patients with perennial allergic rhinitis by inhibiting IgE production after oral ingestion in the heat-inactivated form [29,30]. The action mechanism heat-inactivated form may be different with live bacterial administration. Xiong et al. demonstrated that the combination treatment of IL-4 and IL-10 significantly inhibited TNBS-induced colon tissue damage in mice [31]. IL-4 and IL-10 have anti-inflammatory and immunoregulatory effects and play an important role in the mucosal immune system by inhibiting the synthesis of pro-inflammatory cytokines that alleviate intestinal inflammation [32,33,34,35]. Colitis mice treated with *Trichinella spiralis* extracellular vesicles (Ts-Evs) showed a significant increase in the CD4+IL-4+ cell populations compared to the TNBS group, which was associated with ameliorating colitis by the expansion of Th2 cells [36]. IL-10 inhibits IFN-γ production by Th1 cells and reduces Th17 responses in the DSS model [37,38,39], and mice with IL-10-deficient Treg develop spontaneous colitis [40,41]. In our study, the decrease in IL-4+ and IL-10+ cell frequency in colitis mouse IELs was restored by the administration of *T. halophilus*, demonstrating that inflammation-related cells invading intestinal tissues may be regulated by *T. halophilus*.

Fecal microbiota transplantation has an ameliorative role in UC patients [42], suggesting that microbiota plays a role in the disease. Attenuation of inflammation induced by *T. halophilus* was further validated in the stool microbiome. *Akkermansia muciniphilia* (*A. muciniphilia*), a microbial species identified as a major mucin decomposer of the gut microbiota, was reduced in UC patients [43], and administration of *Lactobacillus reuteri* (*L. reuteri*) to IBD patients may reduce inflammation [44]. In our study, an abundance of *A. muciniphilia* and *L. reuteri* was decreased in DSS-induced colitis mice more than in normal mice, which recovered by *T. halophilus* treatment. In addition, *L. johnsonni* and *Intestinimonas butyriciproducens* were depleted in colitis mice and corrected after treatment with *T. halophilus*. Previous studies have reported significant reductions in genera *Alistipes*, *Barnesiella*, *Oscillibacter*, and *Ruminococcus* observed in IBD [45]. With the same trend, we found *Alistipes*, *Barnesiella*, and *Ruminococcus* were reduced in colitis mice and recovered after administration of *T. halophilus*. *Lactobacillus* performs anti-inflammatory properties by breaking down proinflammatory cytokines [46], and *Bifidobacterium* has an anti-inflammatory function through modification of the SOCS gene [47]. In our study, administration of *T. halophilus* to colitis mice increased the abundance of *Lactobacillus* and *Bifidobacterium*.

CD1d independent NK1.1+CD8+ T cells produced high levels of IFN-γ and TNFα, contributing to colitis pathogenesis, and suggested that DCs and macrophages might be responsible for increasing NK1.1+CD8+ T cells through IL-15 [48]. In active UC, neutrophils have been observed in the crypt epithelium and lamina propria of colonic tissues [49,50], suggesting that neutrophils might play an important role in the pathogenesis of UC. Neutrophil depletion with an anti-Ly6G antibody can ameliorate DSS-induced colitis in rats [51,52]. In this present study, administration of *T. halophilus* affected the frequency of CD11b+Ly6G+ cells in colitis mice and probably had a positive effect on the improvement of intestinal inflammation.

IL-1β is a pro-inflammatory cytokine that plays an integral role in IBD [53,54]. Administration of IL-1β to germ-free mice develops Th17 cells in the small intestine [55], in addition, IL-1β production by commensal bacteria can promote intestinal inflammation [56]. IL-1β was found to be upregulated in active UC patients [57], which activates neutrophils and promotes intestinal pathology [58,59]. Serum concentrations of proinflammatory cytokine TNFα were not different between patients with active or inactive CD or UC, suggesting serum TNFα is not an adequate biomarker for assessing the disease activity in patients with IBD [60]. However, some studies have found that TNFα levels were increased in UC patients and anti-TNFα therapy has promising therapeutic effects [54]. In our study, *T. halophilus* suppresses serum IL-1β and TNFα levels in DSS-induced colitis mice.

Administration of *T. halophilus* may alleviate acute colitis symptoms in mice with DSS-induced colitis, but these experiments were performed in mice fed a standardized diet and the small number of experimental groups may be a limitation of the study. Nevertheless, the current study provides in vivo evidence *T. halophilus* has favorable outcomes against DSS-induced colitis in mice, which may serve as a method for gut microbiome control to complement current therapies for colitis. The anti-inflammatory effects on DSS-induced colitis through the remodeling of the gut microbiota may have been partially immunomodulatory, and therefore further studies are needed to evaluate the immunomodulatory functions of *T. halophilus* in colitis on a large scale.

## Figures and Tables

**Table 1:** Scoring of disease activity index (DAI) of DSS-induced colitis mice.

Score	Weight Loss (W)	Stool Consistency (S)
0	None	Well formed pellets
1	1–5%	
2	5–10%	Loose stool
3	10–20%	
4	>20%	Diarrhea

**Note:** (DAI) = (score of weight loss) + (score of stool consistency).

## Data Availability

The16S rRNA metagenomic data has been deposited in NCBI under the accession number PRJNA772551.

## Supplementary Materials

The following supporting information can be downloaded at: https://www.mdpi.com/article/10.3390/cells11121903/s1, Figure S1: Effects of T. halophilus on activation of dendritic cells in peritoneal macrophage (pMQ) of DSS-induced colitis mice. Figure S2: Frequencies of CD4+ cells in peripheral blood leukocytes (PBL) and spleens of normal control mice, normal mice treated with *T. halophilus*, DSS-induced colitis mice, and colitis mice treated with T. halophilus. Figure S3: Frequencies of CD4+, CD8+, NK1.1+, and CD8+NK1.1+ cells in lymph nodes (LN) of normal control mice, normal mice treated with *T. halophilus*, DSS-induced colitis mice, and colitis mice treated with *T. halophilus*. Table S1: Sources of antibodies used in this work. Table S2: List of primers used in real-time PCR. Table S3: Identification of differences in gut microbiota between normal mice, DSS-induced colitis mice, and *T. halophilus*-treated colitis mice.

## References

[1] Astó E., Méndez I., Audivert S., Farran-Codina A., Espadaler J. (2019). The Efficacy of Probiotics, Prebiotic Inulin-Type Fructans, and Synbiotics in Human Ulcerative Colitis: A Systematic Review and Meta-Analysis. Nutrients.

[2] Kumar N.S.N., Balamurugan R., Jayakanthan K., Pulimood A., Pugazhendhi S., Ramakrishna B.S. (2008). Probiotic administration alters the gut flora and attenuates colitis in mice administered dextran sodium sulfate. J. Gastroenterol. Hepatol..

[3] Berndt B.E., Zhang M., Chen G.-H., Huffnagle G.B., Kao J.Y. (2007). The Role of Dendritic Cells in the Development of Acute Dextran Sulfate Sodium Colitis. J. Immunol..

[4] Zhou L.J., Tedder T.F. (1995). Human blood dendritic cells selectively express CD83, a member of the immunoglobulin superfamily. J. Immunol..

[5] Middel P., Raddatz D., Gunawan B., Haller F., Radzun H.-J. (2006). Increased number of mature dendritic cells in Crohn’s disease: Evidence for a chemokine mediated retention mechanism. Gut.

[6] Eckhardt J., Kreiser S., Döbbeler M., Nicolette C., DeBenedette M.A., Tcherepanova I.Y., Ostalecki C., Pommer A.J., Becker C., Günther C. (2014). Soluble CD83 ameliorates experimental colitis in mice. Mucosal. Immunol..

[7] Ding R.-X., Goh W.-R., Wu R.-N., Yue X.-Q., Luo X., Khine W.W.T., Wu J.-R., Lee Y.-K. (2019). Revisit gut microbiota and its impact on human health and disease. J. Food Drug Anal..

[8] Shen Z.-H., Zhu C.-X., Quan Y.-S., Yang Z.-Y., Wu S., Luo W.-W., Tan B., Wang X.-Y. (2018). Relationship between intestinal microbiota and ulcerative colitis: Mechanisms and clinical application of probiotics and fecal microbiota transplantation. World J. Gastroenterol..

[9] Guo X.Y., Liu X.J., Hao J.Y. (2020). Gut microbiota in ulcerative colitis: Insights on pathogenesis and treatment. J. Dig. Dis..

[10] Szajewska H., Setty M., Mrukowicz J., Guandalini S. (2006). Probiotics in gastrointestinal diseases in children: Hard and not-so-hard evidence of efficacy. J. Pediatr. Gastroenterol. Nutr..

[11] Stagg A.J., Hart A.L., Knight S.C., Kamm M.A. (2003). The dendritic cell: Its role in intestinal inflammation and relationship with gut bacteria. Gut.

[12] Saez-Lara M.J., Gomez-Llorente C., Plaza-Diaz J., Gil A. (2015). The Role of Probiotic Lactic Acid Bacteria and Bifidobacteria in the Prevention and Treatment of Inflammatory Bowel Disease and Other Related Diseases: A Systematic Review of Randomized Human Clinical Trials. BioMed Res. Int..

[13] Kumazawa T., Nishimura A., Asai N., Adachi T. (2018). Isolation of immune-regulatory Tetragenococcus halophilus from miso. PLoS ONE.

[14] Camuesco D., Comalada M., Rodriguez-Cabezas M.E., Nieto A., Lorente M.D., Concha A., Zarzuelo A., Gálvez J. (2004). The intestinal anti-inflammatory effect of quercitrin is associated with an inhibition in iNOS expression. Br. J. Pharmacol..

[15] Couter C.J., Surana N.K. (2016). Isolation and Flow Cytometric Characterization of Murine Small Intestinal Lymphocytes. J. Vis. Exp..

[16] Livak K.J., Schmittgen T.D. (2001). Analysis of relative gene expression data using real-time quantitative PCR and the 2^−ΔΔCT^ Method. Methods.

[17] Perše M., Cerar A. (2012). Dextran Sodium Sulphate Colitis Mouse Model: Traps and Tricks. J. Biomed. Biotechnol..

[18] Eichele D.D., Kharbanda K.K. (2017). Dextran sodium sulfate colitis murine model: An indispensable tool for advancing our understanding of inflammatory bowel diseases pathogenesis. World J. Gastroenterol..

[19] Kim J.A., Yao Z., Perumal V., Kim H.-J., Kim J.H. (2018). Properties of Tetragenococcus halophilus Strains Isolated from Myeolchi (anchovy)-jeotgal. J. Microbiol. Biotechnol..

[20] Marino M., Innocente N., Maifreni M., Mounier J., Cobo-Díaz J.F., Coton E., Carraro L., Cardazzo B. (2017). Diversity within Italian Cheesemaking Brine-Associated Bacterial Communities Evidenced by Massive Parallel 16S rRNA Gene Tag Sequencing. Front. Microbiol..

[21] Christensen H.R., Frøkiær H., Pestka J.J. (2002). Lactobacilli Differentially Modulate Expression of Cytokines and Maturation Surface Markers in Murine Dendritic Cells. J. Immunol..

[22] Lavasani S., Dzhambazov B., Nouri M., Fåk F., Buske S., Molin G., Thorlacius H., Alenfall J., Jeppsson B., Weström B. (2010). A Novel Probiotic Mixture Exerts a Therapeutic Effect on Experimental Autoimmune Encephalomyelitis Mediated by IL-10 Producing Regulatory T Cells. PLoS ONE.

[23] Grosche L., Knippertz I., König C., Royzman D., Wild A.B., Zinser E., Sticht H., Muller Y.A., Steinkasserer A., Lechmann M. (2020). The CD83 Molecule—An Important Immune Checkpoint. Front. Immunol..

[24] Laroux F.S., Grisham M.B. (2001). Immunological basis of inflammatory bowel disease: Role of the microcirculation. Microcirculation.

[25] Amar Y., Rizzello V., Cavaliere R., Campana S., De Pasquale C., Barberi C., Oliveri D., Pezzino G., Costa G., Meddah A.T. (2015). Divergent signaling pathways regulate IL-12 production induced by different species of Lactobacilli in human dendritic cells. Immunol. Lett..

[26] Abdin A.A., Saeid E.M. (2008). An experimental study on ulcerative colitis as a potential target for probiotic therapy by Lactobacillus acidophilus with or without “olsalazine”. J. Crohn’s Colitis.

[27] Keubler L.M., Buettner M., Häger C., Bleich A. (2015). A Multihit Model: Colitis Lessons from the Interleukin-10-deficient Mouse. Inflamm. Bowel Dis..

[28] Berkowitz L., Pardo-Roa C., Ramírez G., Vallejos O.P., Sebastián V.P., Riedel C.A., Álvarez-Lobos M., Bueno S.M. (2019). The absence of interleukin 10 affects the morphology, differentiation, granule content and the production of cryptidin-4 in Paneth cells in mice. PLoS ONE.

[29] Masuda S., Yamaguchi H., Kurokawa T., Shirakami T., Tsuji R., Nishimura I. (2008). Immunomodulatory effect of halophilic lactic acid bacterium Tetragenococcus halophilus Th221 from soy sauce moromi grown in high-salt medium. Int. J. Food Microbiol..

[30] Nishimura I., Igarashi T., Enomoto T., Dake Y., Okuno Y., Obata A. (2009). Clinical Efficacy of Halophilic Lactic Acid Bacterium Tetragenococcus halophilus Th221 from Soy Sauce Moromi for Perennial Allergic Rhinitis. Allergol. Int..

[31] Xiong J., Lin Y.-H., Bi L.-H., Wang J.-D., Bai Y., Liu S.-D. (2013). Effects of Interleukin-4 or Interleukin-10 gene therapy on trinitrobenzenesulfonic acid-induced murine colitis. BMC Gastroenterol..

[32] Braat H., Peppelenbosch M.P., Hommes D.W. (2003). Interleukin-10-based therapy for inflammatory bowel disease. Expert Opin. Biol. Ther..

[33] Li M.C., He S.H. (2004). IL-10 and its related cytokines for treatment of inflammatory bowel disease. World J. Gastroenterol..

[34] Iijima H., Takahashi I., Kishi D., Kim J.-K., Kawano S., Hori M., Kiyono H. (1999). Alteration of Interleukin 4 Production Results in the Inhibition of T Helper Type 2 Cell–Dominated Inflammatory Bowel Disease in T Cell Receptor α Chain–Deficient Mice. J. Exp. Med..

[35] Khan M.M., Chatterjee S., Dwivedi V.P., Pandey N.K., Singh Y., Tousif S., Bhavesh N.S., Van Kaer L., Das J., Das G. (2012). CD4+ T Cell-derived Novel Peptide Thp5 Induces Interleukin-4 Production in CD4+ T Cells to Direct T Helper 2 Cell Differentiation. J. Biol. Chem..

[36] Yang Y., Liu L., Liu X., Zhang Y., Shi H., Jia W., Zhu H., Jia H., Liu M., Bai X. (2020). Extracellular Vesicles Derived From Trichinella spiralis Muscle Larvae Ameliorate TNBS-Induced Colitis in Mice. Front. Immunol..

[37] Tomoyose M., Mitsuyama K., Ishida H., Toyonaga A., Tanikawa K. (1998). Role of Interleukin-10 in a Murine Model of Dextran Sulfate Sodium-Induced Colitis. Scand. J. Gastroenterol..

[38] Rodrigues V.F., Bahia M.P.S., Cândido N.R., Moreira J.M.P., Oliveira V.G., Araújo E.S., Rodrigues Oliveira J.L., Rezende M.C., Correa A., Negrão-Corrêa D. (2018). Acute infection with Strongyloides venezuelensis increases intestine production IL-10, reduces Th1/Th2/Th17 induction in colon and attenuates Dextran Sulfate Sodium-induced colitis in BALB/c mice. Cytokine.

[39] Powrie F., Leach M.W., Mauze S., Menon S., Caddle L.B., Coffman R.L. (1994). Inhibition of Th1 responses prevents inflammatory bowel disease in scid mice reconstituted with CD45RBhi CD4+ T cells. Immunity.

[40] Rubtsov Y.P., Rasmussen J.P., Chi E.Y., Fontenot J., Castelli L., Ye X., Treuting P., Siewe L., Roers A., Henderson W.R. (2008). Regulatory T Cell-Derived Interleukin-10 Limits Inflammation at Environmental Interfaces. Immunity.

[41] Chaudhry A., Samstein R., Treuting P., Liang Y., Pils M.C., Heinrich J.-M., Jack R.S., Wunderlich F.T., Brüning J.C., Muller W. (2011). Interleukin-10 Signaling in Regulatory T Cells Is Required for Suppression of Th17 Cell-Mediated Inflammation. Immunity.

[42] Tian Y., Zhou Y., Huang S., Li J., Zhao K., Li X., Wen X., Li X.-A. (2019). Fecal microbiota transplantation for ulcerative colitis: A prospective clinical study. BMC Gastroenterol..

[43] Geerlings S.Y., Kostopoulos I., De Vos W.M., Belzer C. (2018). Akkermansia muciniphila in the Human Gastrointestinal Tract: When, Where, and How?. Microorganisms.

[44] Wang H., Zhou C., Huang J., Kuai X., Shao X. (2020). The potential therapeutic role of Lactobacillus reuteri for treatment of inflammatory bowel disease. Am. J. Transl. Res..

[45] Knox N.C., Forbes J.D., Van Domselaar G., Bernstein C.N. (2019). The Gut Microbiome as a Target for IBD Treatment: Are We There Yet?. Curr. Treat Options Gastroenterol..

[46] von Schillde M.-A., Hörmannsperger G., Weiher M., Alpert C.-A., Hahne H., Bäuerl C., van Huynegem K., Steidler L., Hrncir T., Pérez-Martínez G. (2012). Lactocepin Secreted By Lactobacillus Exerts Anti-Inflammatory Effects By Selectively Degrading Proinflammatory Chemokines. Cell Host Microbe.

[47] Okada Y., Tsuzuki Y., Hokari R., Komoto S., Kurihara C., Kawaguchi A., Nagao S., Miura S. (2009). Anti-inflammatory effects of the genus Bifidobacterium on macrophages by modification of phospho-I kappaB and SOCS gene expression. Int. J. Exp. Pathol..

[48] Lee S.W., Park H.J., Cheon J.H., Wu L., Van Kaer L., Hong S. (2018). iNKT Cells Suppress Pathogenic NK1.1+CD8+ T Cells in DSS-Induced Colitis. Front. Immunol..

[49] Kishida K., Kohyama M., Kurashima Y., Kogure Y., Wang J., Hirayasu K., Suenaga T., Kiyono H., Kunisawa J., Arase H. (2015). Negative regulation of DSS-induced experimental colitis by PILRα. Int. Immunol..

[50] Naito Y., Takagi T., Yoshikawa T. (2007). Neutrophil-Dependent Oxidative Stress in Ulcerative Colitis. J. Clin. Biochem. Nutr..

[51] Munyaka P.M., Rabbi M.F., Khafipour E., Ghia J.-E. (2016). Acute dextran sulfate sodium (DSS)-induced colitis promotes gut microbial dysbiosis in mice. J. Basic Microbiol..

[52] Natsui M., Kawasaki K., Takizawa H., Hayashi S.-I., Matsuda Y., Sugimura K., Seki K., Narisawa R., Sendo F., Asakura H. (1997). Selective depletion of neutrophils by a monoclonal antibody, RP-3, suppresses dextran sulphate sodium-induced colitis in rats. J. Gastroenterol. Hepatol..

[53] Mao L., Kitani A., Strober W., Fuss I.J. (2018). The Role of NLRP3 and IL-1β in the Pathogenesis of Inflammatory Bowel Disease. Front. Immunol..

[54] Sands B.E., Kaplan G.G. (2007). The role of TNFalpha in ulcerative colitis. J. Clin. Pharmacol..

[55] Shaw M.H., Kamada N., Kim Y.-G., Núñez G. (2012). Microbiota-induced IL-1β, but not IL-6, is critical for the development of steady-state TH17 cells in the intestine. J. Exp. Med..

[56] Seo S.-U., Kamada N., Muñoz-Planillo R., Kim Y.-G., Kim D., Koizumi Y., Hasegawa M., Himpsl S.D., Browne H., Lawley T.D. (2015). Distinct Commensals Induce Interleukin-1β via NLRP3 Inflammasome in Inflammatory Monocytes to Promote Intestinal Inflammation in Response to Injury. Immunity.

[57] Ranson N., Veldhuis M., Mitchell B., Fanning S., Cook A.L., Kunde D., Eri R. (2018). NLRP3-Dependent and -Independent Processing of Interleukin (IL)-1β in Active Ulcerative Colitis. Int. J. Mol. Sci..

[58] Coccia M., Harrison O.J., Schiering C., Asquith M.J., Becher B., Powrie F., Maloy K.J. (2012). IL-1β mediates chronic intestinal inflammation by promoting the accumulation of IL-17A secreting innate lymphoid cells and CD4+ Th17 cells. J. Exp. Med..

[59] Hall L.J., Murphy C.T., Quinlan A., Hurley G., Shanahan F., Nally K., Melgar S. (2013). Natural killer cells protect mice from DSS-induced colitis by regulating neutrophil function via the NKG2A receptor. Mucosal Immunol..

[60] Avdagić N., Babić N., Šeremet M., Delić-Šarac M., Drače Z., Denjalić A., Nakaš-Ićindić E. (2013). Tumor necrosis factor-alpha serum level in assessment of disease activity in inflammatory bowel diseases. Med. Glas..

