# Supplementary material for: Tetragenococcus halophilus Alleviates Intestinal Inflammation in Mice by Altering Gut Microbiota and Regulating Dendritic Cell Activation via CD83
Source: Cells. 2022 Jun 12;11(12):1903. doi: 10.3390/cells11121903 (PMC9221263; doi:10.3390/cells11121903)
Supplement: Supplementary file 1 [file cells-11-01903-s001.zip › Supplementary Figures S1¿CS3 and Tables S1¿CS2.pdf]

Supplementary Figure S1

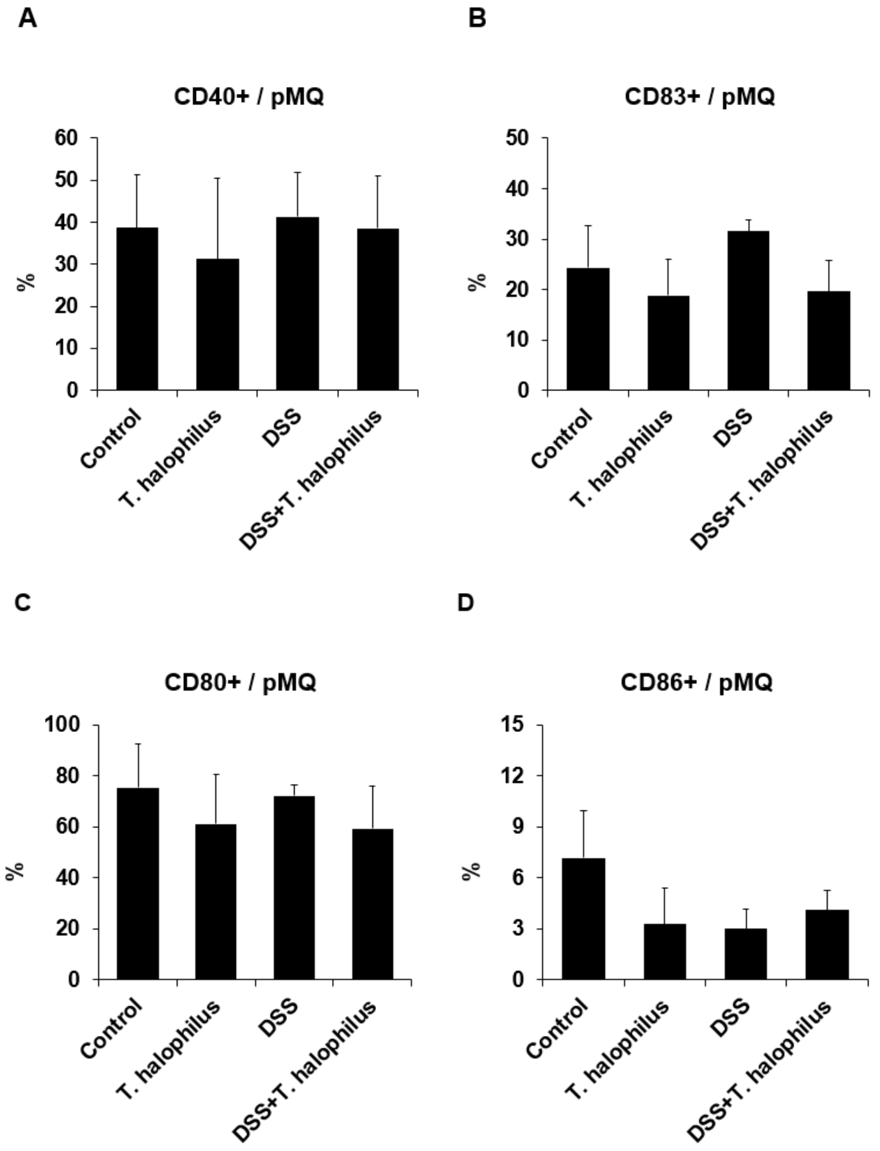

Supplementary Figure S1. Effects of *T. halophilus* on activation of dendritic cells in peritoneal macrophage (pMQ) of DSS-induced colitis mice. Frequencies of cells positive for DCs costimulatory molecules CD40, CD83, CD80, and CD86 in pMQ were assessed in normal control mice, normal mice treated with *T. halophilus* (4.8×10^8 CFU/mouse/day), DSS-induced colitis mice, and colitis mice treated with *T. halophilus* (4.8×10^8 CFU/mouse/day) (**A**–**D**). Isolated pMQ was evaluated by flow cytometry surface staining. The number of mice used for experiments was four in each group. Experiments were performed independently at least twice.

Supplementary Figure S2

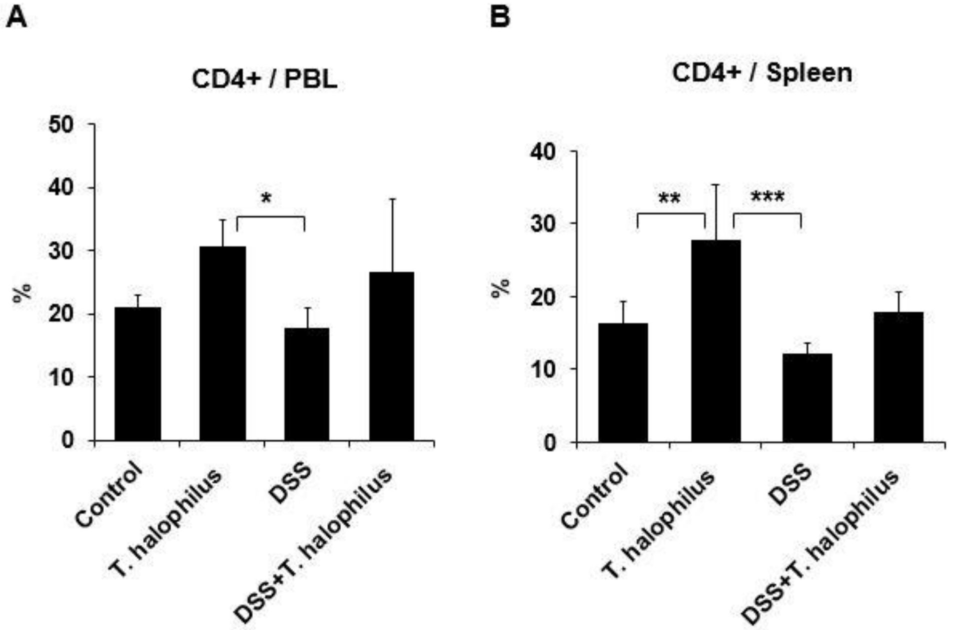

Supplementary Figure S2. Frequencies of CD4+ cells in peripheral blood leukocytes (PBL) and spleens of normal control mice, normal mice treated with *T. halophilus*, DSS-induced colitis mice, and colitis mice treated with *T. halophilus* were evaluated by FACS analysis (**A**,**B**). The p-value was determined by one-way ANOVA followed by Bonferroni's correction with GraphPad. Four mice were used in each experimental group. *p*-values significantly different are indicated by asterisk as follows: *, $p < 0.05$; **, $p < 0.01$; ***, $p < 0.001$.

Supplementary Figure S3

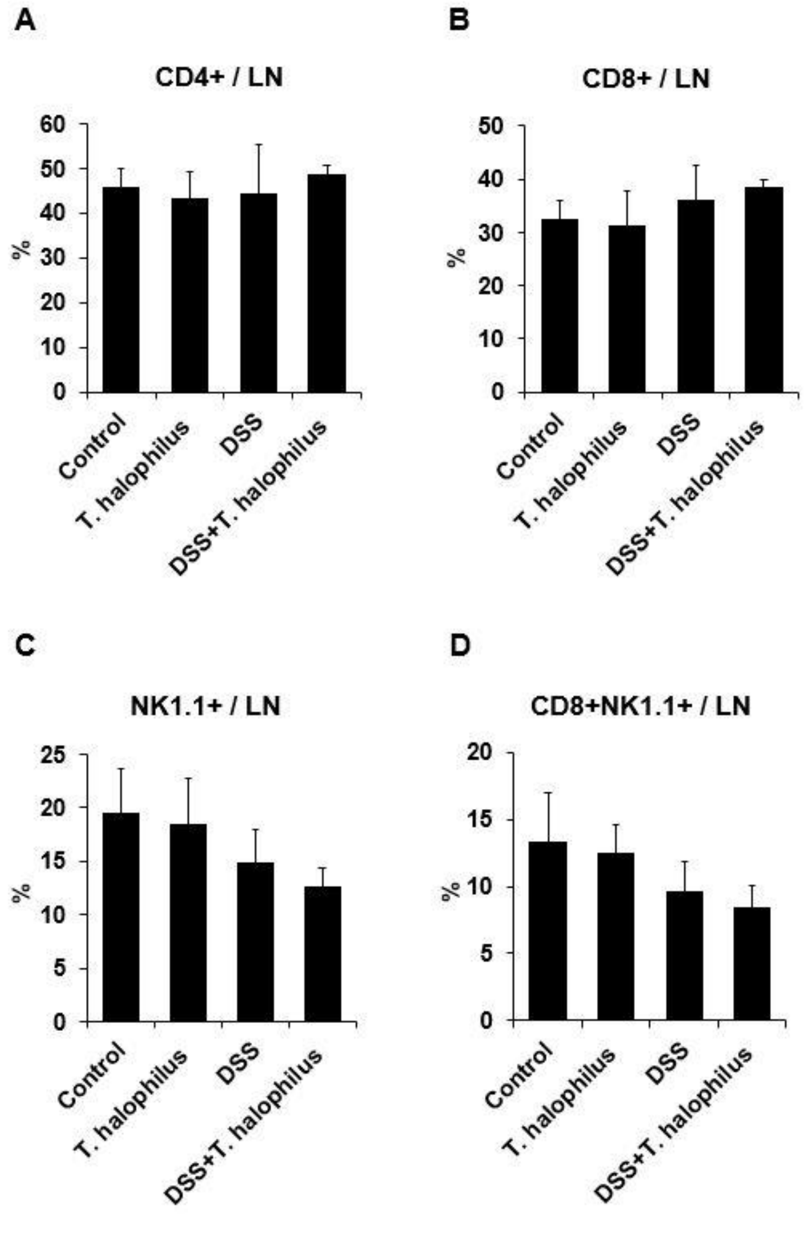

Supplementary Figure S3. Frequencies of CD4+, CD8+, NK1.1+, and CD8+NK1.1+ cells in lymph nodes (LN) of normal control mice, normal mice treated with *T. halophilus*, DSS-induced colitis mice, and colitis mice treated with *T. halophilus* were evaluated by FACS analysis (**A–D**). Four mice were used in each experimental group. Experiments were performed independently at least twice.

Supplementary Table S1. Sources of antibodies used in this work.

FACS markers	Fluorochromes	Company
**Surface staining markers**		
CD40	PerCP-eFluro 710	eBioscience, San Diego, CA, USA
CD83	eFluro 660	eBioscience, San Diego, CA, USA
CD80	PE-Cyanine7	eBioscience, San Diego, CA, USA
CD86	FITC	eBioscience, San Diego, CA, USA
CD4	PE-Cy7	eBioscience, San Diego, CA, USA
CD4	FITC	eBioscience, San Diego, CA, USA
CD8	PerCP-Cyanine 5.5	eBioscience, San Diego, CA, USA
CD11c	PE-Cyanine7	eBioscience, San Diego, CA, USA
CD11b	PE	eBioscience, San Diego, CA, USA
Ly6G	FITC	eBioscience, San Diego, CA, USA
NK1.1	PE-Cyanine7	eBioscience, San Diego, CA, USA
**Intracellular staining markers**		
IL-4	FITC	Invitrogen, Carlsbad, CA, USA
IL-10	APC	Invitrogen, Carlsbad, CA, USA

Supplementary Table S2. List of primers used in real-time PCR.

Gene Name	Forward	Reverse
NLRP3	GGTCCTAGCCTCGTCACCAT	AGAAAGCCATTAACACAATCAGATG
IL-1β	GCAACTGTTCCTGAACTCAACT	ATCTTTTGGGGTCCGTCCAACT
TNFα	ATGGCCTCCCTCTCATCAGT	TTGCTACGACGTGGGCTAC
CCR7	AACCCAGGAAAAACGTGCTG	CCGTGGTATTCTCGCCGATG
β-actin	TGTCCACCTTCCAGCAGATGT	AGCTCAGTA ACAGTCCGCCTAG
